# Mechanical and Tribological Properties of Laminated (NbTaMoW)N*_x_* Films

**DOI:** 10.3390/ma17204962

**Published:** 2024-10-11

**Authors:** Yan-Zhi Liao, Yung-I Chen

**Affiliations:** Department of Optoelectronics and Materials Technology, National Taiwan Ocean University, Keelung 202301, Taiwan; 11289034@mail.ntou.edu.tw

**Keywords:** laminated films, mechanical properties, tribological properties

## Abstract

Laminated (NbTaMoW)N*_x_* films were prepared via co-sputtering. The sputtering variables were a substrate holder rotation speed of 2 and 10 rpm and a nitrogen flow ratio (*f*_N2_ = N_2_/(Ar + N_2_)) of 0.1, 0.2, and 0.4. The (NbTaMoW)N*_x_* films fabricated at 30 rpm displayed columnar structures. The phase structures of the laminated (NbTaMoW)N*_x_* films varied from multiple body-centered cubic phases to a nanocrystalline and a face-centered cubic phase as the *f*_N2_ increased from 0.1 to 0.2 and 0.4. The mechanical and tribological properties of the laminated (NbTaMoW)N*_x_* films were evaluated. The laminated (NbTaMoW)N*_x_* films deposited at an *f*_N2_ of 0.4 had hardnesses of 25.2 and 26.1 GPa when prepared at 2 and 10 rpm, respectively, lower than the value of 29.9 GPa for the columnar (NbTaMoW)N*_x_* film prepared at an *f*_N2_ of 0.4 and 30 rpm. In contrast, the wear resistances of the laminated (NbTaMoW)N_*x*_ films were superior to those of the columnar (NbTaMoW)N*_x_* films.

## 1. Introduction

High-entropy alloys (HEAs) [1], also mentioned as multi-principal-element alloys [2], have attracted researchers’ attention for enhancing the characteristics of alloy materials. Refractory high-entropy alloys consisting of group IVB–VIB elements have been extensively explored to achieve innovative developments to respond to increasingly demanding and versatile industrial environments. The high-entropy effect causes these multi-principal-element alloys to form solid solutions with randomly distributed elements, and their films exhibit excellent mechanical, anticorrosive, and antioxidative properties [3]. The rapid quenching in the thin film process avoids phase transformation, favoring solid solution formation [4]. Solid solution and grain-boundary-strengthening mechanisms have improved HEA films’ mechanical properties, correlating with those of their bulk formats [5,6]. NbMoTaW [7,8], TaNbHfZr [9], and TiZrHfTa [10] alloys, typical refractory multi-principal-element alloys forming a body-centered cubic (BCC) phase, have hardness values of 3–5 GPa; however, their films exhibit distinct elevations in hardness. NbMoTaW films have high hardness values of 12–16 GPa [11,12,13]. The hardness of TaNbHfZr films correlates with their structure, e.g., amorphous TaNbHfZr films prepared at room temperature and 500 °C exhibit hardness values of 8 and 13 GPa [6,14], respectively. In contrast, crystalline TaNbHfZr fabricated at 700 °C has a high hardness of 15 GPa [6], while the hardness of TiZrHfTa films is relatively low, at 5–6 GPa [15]. Developing high-entropy alloy nitride (HEAN) films by incorporating nitrogen into HEA films offers a practical pathway to enhance mechanical properties. The (NbMoTaW)N*_x_* and (TiZrHfTa)N*_x_* films have hardness values of 25–32 [16] and 14–26 GPa [15], respectively. HEAN films are expected to serve as hard coating layers, corrosion-resistant layers [17,18], and diffusion barrier layers with improved mechanical performance [19,20,21].

By extension, the mechanical characteristics of surface-modified materials need to include an excellent hardness and elastic modulus, a low coefficient of friction (COF), and good wear-resistant performance in order to be applied in industrial tools. The development of bilayer and multilayer films combines the advantage of sublayers, representing an alternative approach for enhancing thin films’ properties [22]. Moreover, nano-multilayer films not only enhance the hardness of coatings but also improve their toughness and resistance to crack propagation, as seen in hard-yet-tough coatings [23]. Multilayer and nanocomposite films with a nanoscale structure have been proposed for combining high hardness and toughness [24]. In our previous studies [25,26], laminated thin films with cyclical gradient concentration deposition were developed through co-sputtering using multiple targets under a low substrate holder rotation speed (*R_H_*). (NbTaMoW)N*_x_* films with columnar structures and good mechanical and anticorrosive properties were developed in our previous study [16]; therefore, exploring the characteristics of laminated (NbTaMoW)N*_x_* films becomes interesting. Forming a laminated structure to obtain films with hard and tough properties is possible. This study conducted co-sputtering processes using four pure element targets, Nb, Ta, Mo, and W, in an N_2_ and Ar gas mixture with various gas ratios. The substrate holder’s rotation speeds were adjusted to 2 and 10 rpm to perform cyclical gradient concentration deposition and form the laminated films [11]. The crystalline structures of the laminated (NbTaMoW)N*_x_* films were investigated, and their mechanical and tribological properties were examined.

## 2. Materials and Methods

This study utilized co-sputtering equipment with four sputter guns, as described in [11]. Different direct current (DC) powers were applied to these sputter guns with targets 50.8 mm in diameter. The film substrates included Si wafers and SUS420 plates preheated to 400 °C. The Ta interlayer and laminated (NbTaMoW)N*_x_* films were deposited at substrate holder rotation speeds (*R_H_*) of 10 and 2 rpm, represented as batches B and C, respectively. The Ta interlayer was deposited at a 200 W DC power and a 20 sccm Ar flow for 10 min. Subsequently, DC powers of 160, 200, 200, and 120 W were applied to Nb, Ta, Mo, and W targets, respectively. The nitrogen flow ratio (*f*_N2_ = [N_2_/(N_2_ + Ar)]) was adjusted to 0.1, 0.2, and 0.4 under a total flow rate of 30 sccm to deposit the (NbTaMoW)N*_x_* films. The deposition time was 50 min. The batch B samples prepared at an *R_H_* of 10 rpm were designated as B01, B02, and B04 when *f*_N2_ levels were set at 0.1, 0.2, and 0.4, respectively, whereas the batch C samples prepared at an *R_H_* of 2 rpm were designated as C01, C02, and C04 when *f*_N2_ levels were set at 0.1, 0.2, and 0.4, respectively. Samples A01, A02, and A04 prepared at an *R_H_* of 30 rpm in our previous study [16] were added to this article for comparison (Table 1).

The films’ phases were identified using an X-ray diffractometer (XRD, X’Pert PRO MPD, PANalytical, Almelo, The Netherlands) with Cu Kα radiation and an incidence angle of 1°. The nanostructures were examined using transmission electron microscopy (TEM, JEM-2010E, JEOL, Tokyo, Japan). The mechanical properties, hardness, and elastic modulus of the films were measured using a nanoindentation tester (TI-900 Triboindenter, Hysitron, Minneapolis, MN, USA) equipped with a Berkovich diamond probe tip [27]. The elastic modulus was calculated using the Oliver–Pharr method [28] and a Poisson ratio of 0.25. The residual stress on the films was determined using the curvature method [29].

(1) $$\sigma_{f}t_{f}=\frac{E_{S}{h_{S}}^{2}}{6(1-\nu_{S})R_{f}}$$

where *σ_f_* is the residual stress, *t_f_* is the film thickness, *E_S_* is the elastic modulus (130.2 GPa), *ν_S_* is the Poisson’s ratio (0.279) [30], *h_S_* is the thickness (525 μm) of the Si substrate, and *R_f_* is the measured radius of curvature. A scratch tester (RST3, Anton Paar, Graz, Austria) was used to assess the adhesion of films to SUS420 substrates. A vertical load was applied using a diamond tip with a diameter of 200 μm moving in a straight line at 5 mm/min. A tribometer (TRB^3^, Anton Paar, Graz, Austria) was exploited to examine films’ wear resistance using the pin-on-disk method. The wear tests were performed using a tungsten carbide (WC, 6 wt% Co) ball 6 mm in diameter as the counterface; these tests were completed using a load of 1 N, a tangential velocity of 104.5 mm/s, a sliding distance of 100 m, and a track diameter of 16 mm. Three-dimensional images and two-dimensional profiles of the tested samples were obtained using a white-light interferometer (Profilm3D, Filmetrics, San Diego, CA, USA).

## 3. Results and Discussion

### 3.1. Phase Structure

**Figure 1:**
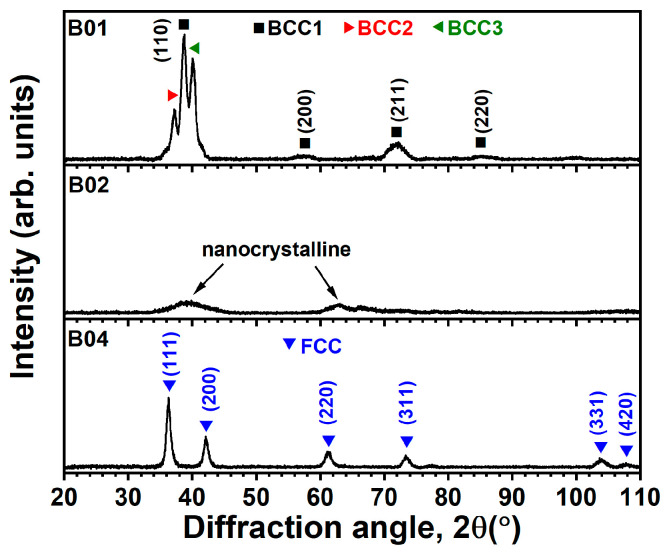
XRD patterns of the laminated (NbTaMoW)N*_x_* films fabricated using an *R_H_* of 10 rpm.

**Figure 2:**
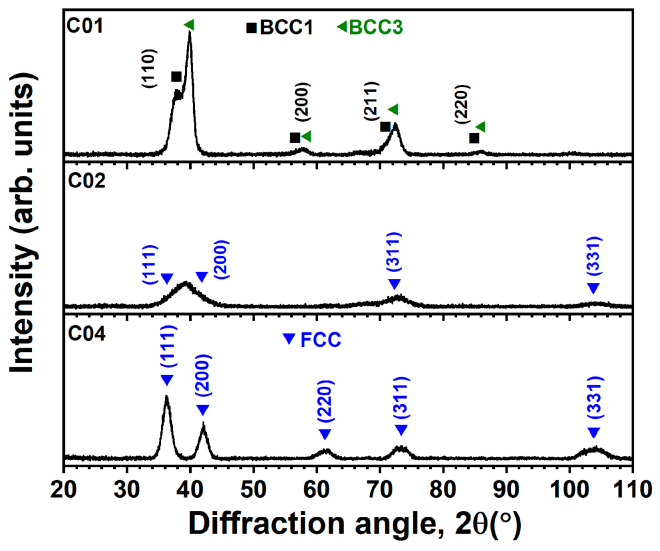
XRD patterns of the laminated (NbTaMoW)N*_x_* films fabricated using an *R_H_* of 2 rpm.

Figure 1 and Figure 2 display the XRD patterns of the (NbTaMoW)N*_x_* films prepared with an *R_H_* of 10 and 2 rpm, respectively. The structures exhibit evident variation when altering the *f*_N2_ value from 0.1 to 0.2 and 0.4. The B01 and C01 samples exhibit multiple BCC phases, the B02 and C02 films reveal nanocrystalline structures with broad reflections, and the B04 and C04 films have a face-centered cubic (FCC) phase. The nanocrystalline C02 seems to have an FCC phase. In our previous study [16], the (NbTaMoW)N*_x_* films fabricated using an *R_H_* of 30 rpm exhibited chemical compositions of (Nb_0.16_Ta_0.29_Mo_0.32_W_0.23_)N_0.07_ (A01), (Nb_0.14_Ta_0.29_Mo_0.31_W_0.26_)N_0.19_ (A02), and (Nb_0.11_Ta_0.25_Mo_0.33_W_0.31_)N_0.49_ (A04) when prepared at an *f*_N2_ of 0.1 to 0.2 and 0.4, respectively. The phases were dominated by multiple BCC, M_2_N-type FCC, and MN-type FCC for A01, A02, and A04 samples, respectively. The NbTaMoW alloys consist of group VB and VIB elements and form a BCC solid solution because the alloys’ valence electron concentrations are in the range of 4.18–6.87 [31,32]. N atoms occupy the interstitials in metallic alloy structures. The N-containing HEAN films could form an amorphous or nanocrystalline structure when the N content surpasses the solubility in a BCC structure but is not enough to create an FCC phase, such as in (CrHfMoTaW)N*_x_* [33] and (HfNbTiVZr)N*_x_* [34] films.

**Figure 3:**
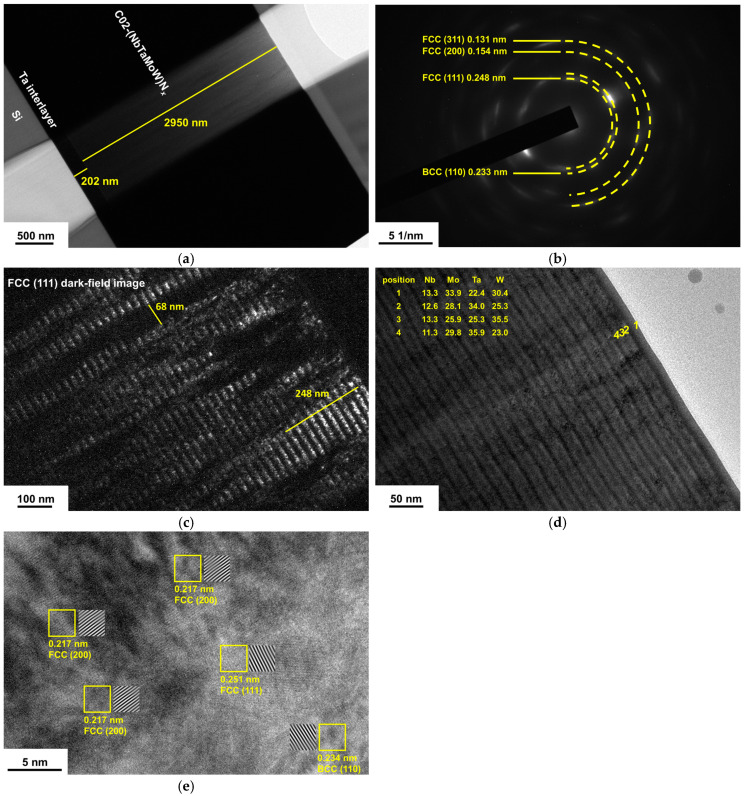
(**a**) XTEM image and (**b**) SAED pattern of the C02 film; (**c**) dark field image and (**d**) EDS results of the surficial region of the C02 film; (**e**) HRTEM image of the C02 film.

**Figure 4:**
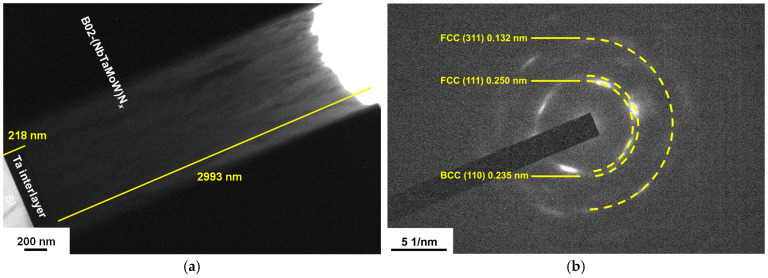

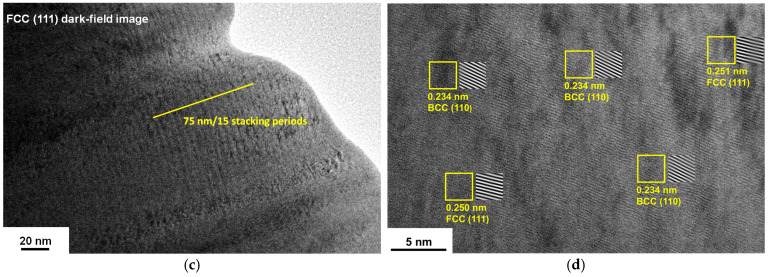
(**a**,**c**) XTEM image, (**b**) SAED pattern, and (**d**) HRTEM image of the B02 film.

Figure 3a exhibits a cross-sectional TEM (XTEM) observation of the C02 sample. The C02 film and Ta interlayer thicknesses were 2950 and 202 nm, respectively. Figure 3b exhibits the selected area electron diffraction (SAED) pattern of the C02 film, which displays *d*-spacing values of 0.233 nm relating to a BCC (110) plane and 0.248, 0.154, and 0.131 nm relating to FCC (111), (220), and (311) planes, respectively. Figure 3c depicts a dark-field image corresponding to the diffraction signal of FCC (111) planes, revealing an evident laminated structure caused by a cyclical gradient concentration with a stacking period of 25 nm. Because the deposition time was 50 min, the *R_H_* for batch C samples was calibrated at 2.36 rpm. Figure 3d depicts the EDS analysis of the C02 films. Points 2 and 4 exhibit Ta- and Mo-enriched compositions, whereas point 3 exhibits a W-enriched composition. Figure 3e depicts a high-resolution TEM (HRTEM) image of the C02 film. The lattice fringes indicate that the C02 film is crystalline and comprises BCC and FCC mixed phases. Figure 4a displays an XTEM image of the B02 sample with a 218 nm thick Ta interlayer and a 2993 nm thick B02 film. Figure 4b shows the SAED pattern of the B02 sample, which exhibits *d*-spacing values of 0.235 nm relating to the BCC (110) plane and 0.250 and 0.132 nm relating to the FCC (111) and (311) planes, respectively. Figure 4c shows a dark-field image, which reveals an evident laminated structure caused by cyclical gradient concentration, and the stacking period of approximately 5.0 nm is narrower than that of the C02 sample. Figure 4d shows an HRTEM image of the B02 film, which indicates crystallinity.

### 3.2. Mechanical Properties

**Figure 5:**
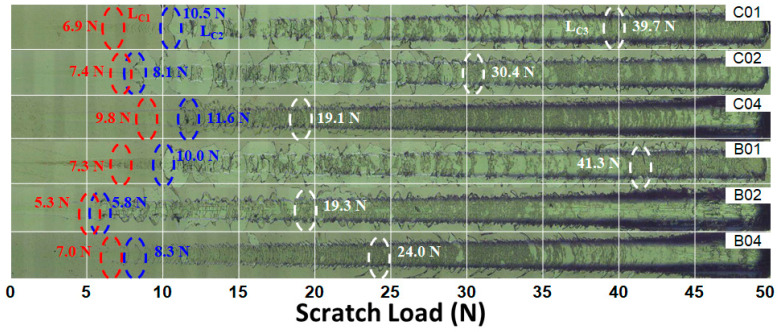
Scratch morphologies of laminated (NbTaMoW)N*_x_* films.

**Figure 6:**
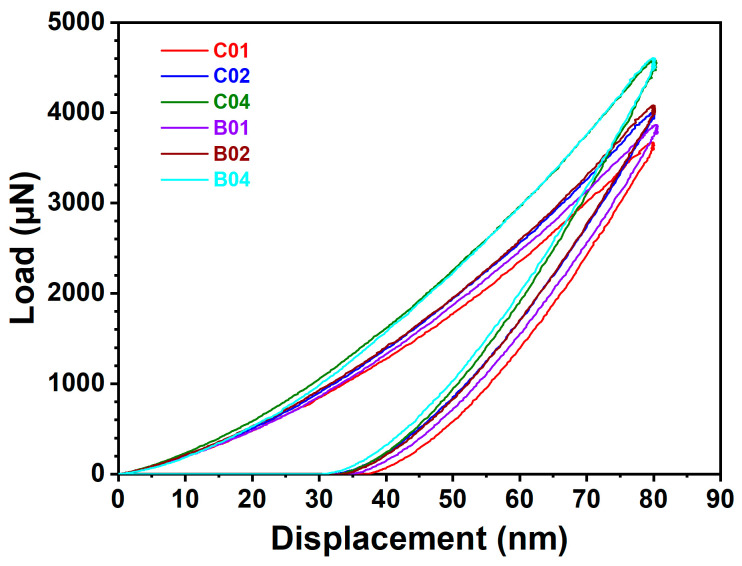
Load–displacement curves of laminated (NbTaMoW)N*_x_* films.

The adhesion between the laminated (NbTaMoW)N*_x_* films and SUS420 substrates was assessed using the scratch test, with the scratch load incrementally increasing from 0.5 to 50 N. Figure 5 displays the surface morphologies of the scratched (NbTaMoW)N*_x_* films. As the N content increases, the surface morphology transforms from buckling and wedging-type failures to continuous conformal cracking with slight recovery-type failures. The scratch tracks indicate three critical loads for film delamination, namely, *L*_C1_, *L*_C2_, and *L*_C3_ [35], signifying the initial crack and delamination for films and the exposure of the substrate. Chipping and conformal cracks were initially observed at a critical load *L*_C1_ of approximately 5–10 N. *L*_C2_ corresponds to localized adhesive failure, with a load of roughly 6–12 N. The *L*_C3_ values vary significantly, decreasing from 40–41 N for the C01 and B01 samples to 19 N for the C04 and B02 samples. The high adhesion strength of the samples prepared at an *f*_N2_ of 0.1 is attributed to the fact that these films predominantly consist of metallic phases, which provide higher ductility. Figure 6 depicts the load–unloading plots of the indentation tests for the laminated (NbTaMoW)N*_x_* films. The indentation depth is set at 80 nm. These curves seem smooth without discontinuous variations contributed by phase transformation during the tests [36]. Table 2 shows the mechanical properties of the columnar and laminated (NbTaMoW)N*_x_* films. The films with high N contents, prepared with higher *f*_N2_ values, had higher mechanical properties. The hardness and elastic modulus increased from 21.6 and 325 GPa for the B01 film to 23.0 and 337 GPa for the B02 film, and 26.1 and 377 GPa for the B04 film, respectively. A similar variation tendency was observed for the batch C samples. The hardness and elastic modulus increased from 19.3 and 317 GPa for the C01 film to 23.4 and 337 GPa for the C02 film and 25.2 and 386 GPa for the C04 film, respectively. However, the films fabricated at a lower *R_H_* exhibited lower hardness values. The hardness decreased from 29.9 GPa for the A04 sample fabricated at 30 rpm to 26.1 GPa for the B04 sample (*R_H_* of 10 rpm), and further reduced to 25.2 GPa for the C04 sample (*R_H_* of 2 rpm). The hardness of 29.9 GPa is equivalent to the reported value of 30–30.8 GPa for (MoNbTaVW)N*_x_* [37] and (NbTaMoW)N*_x_* [38] films. The increase in *R_H_* decreased the equilibrated laminated period of the Ru–Zr multilayer films [26], which improved the mechanical properties. The laminated films’ hardness and elastic modulus increased with the increase in the inlet *f*_N2_ during the sputtering process, and the films’ residual stress exhibited a similar trend. In contrast, the elastic modulus increased from 343 GPa for the A04 sample to 377 GPa for the B04 sample and 386 GPa for the C04 sample. This increase in elastic modulus with increased equilibrated laminated period should be ascribed to the strong bonding strength of the nitride. Previous studies [37,38,39] pointed out that the hardnesses of HEAN films increases with the rising N content in the films or the use of a nitrogen flow. The common characteristic of these HEAN films with the highest hardness was the forming of an FCC phase. When nitrogen is introduced into NbTaMoW films, the mechanical properties are improved due to the nitride phase formation and the transition of the bonding structure from metallic to covalent. The residual stress increased from −0.04 to −0.18 and −1.91 GPa, accompanied by an increase in hardness from 21.6 to 23.0 and 26.1 GPa for the B01, B02, and B03 samples, respectively. A similar trend was observed for the hardness values of C01, C02, and C03 samples. This result implies the influence of laminated structure formation.

### 3.3. Tribological Properties

**Figure 7:**
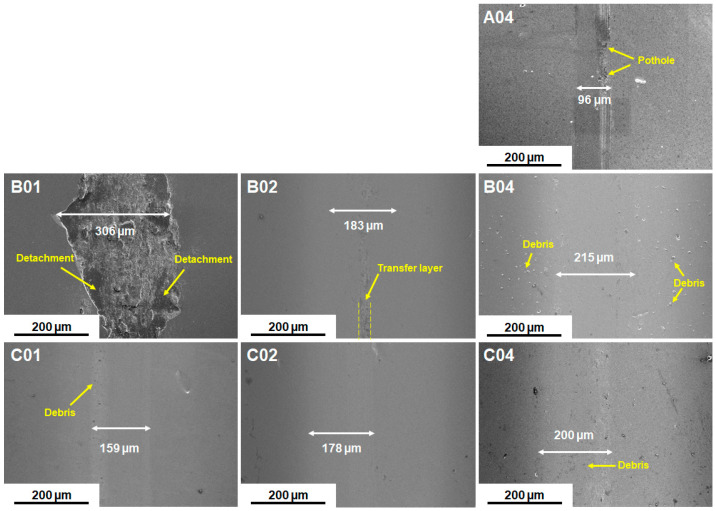
Wear scars of (NbTaMoW)N*_x_* films after the wear test.

**Figure 8:**
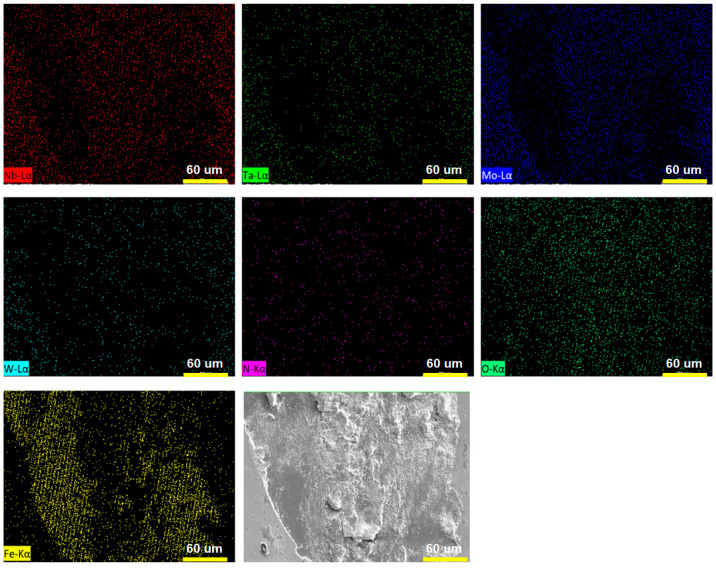
Elemental mappings of partially detached B01 films.

**Figure 9:**
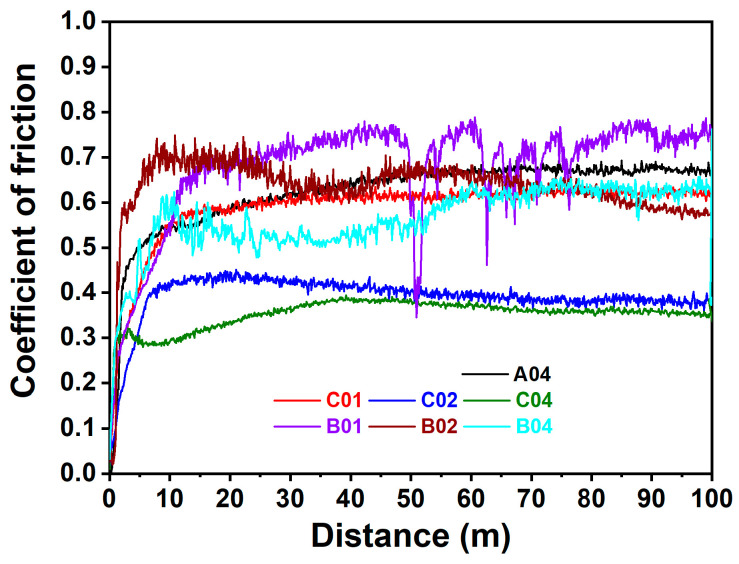
COF variations of (NbTaMoW)N*_x_* films during the wear tests.

**Figure 10:**
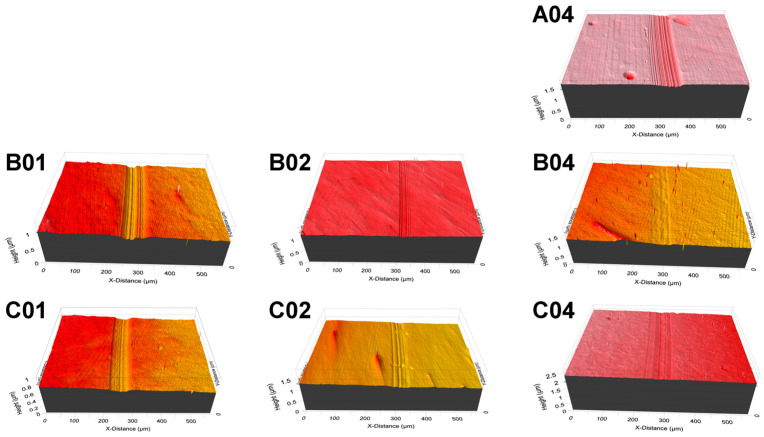
Three-dimensional images of (NbTaMoW)N*_x_* films’ wear tracks.

**Figure 11:**
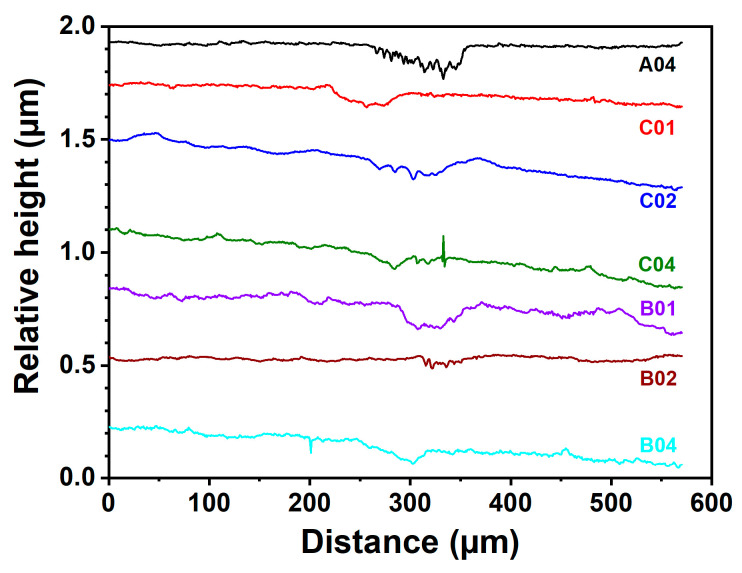
Two-dimensional profiles of (NbTaMoW)N*_x_* films’ wear tracks.

Wear tests were conducted on the (NbTaMoW)N*_x_* films deposited on SUS 420 stainless steel. Figure 7 presents the scanning electron microscope images of the (NbTaMoW)N*_x_* films after undergoing wear tests. The columnar A04 film exhibited a narrower wear width of 96 µm relating to 59–215 µm for the laminated (NbTaMoW)N*_x_* films. Potholes were observed on localized regions of the A04 film; however, these potholes were not worn through. Debris and transfer layers were the most significant forms of damage on the worn surface of the laminated (NbTaMoW)N*_x_* films. A severe detachment was observed for the B01 film. Figure 8 displays a magnified image and EDS analyses of the partially detached B01 film. Parts of the B01 film exhibited Fe signals, indicating the SUS420 substrate’s exposure. In contrast, the rest of the regions of the B01 film revealed a smooth surface similar to the other laminated films. Except for the B01 film, all laminated (NbTaMoW)N*_x_* films exhibited smooth surfaces in the interior portions of the wear tracks. Most of the debris was implanted into the surfaces. In contrast, the debris from the B04 film was distributed outside the wear track and on the free surface, which could be attributed to the higher hardness of the B04 film. Figure 9 displays the record of the COF in a wear distance of 0–100 m. Except for the B01 sample, all the other films displayed a stable COF after the running-in period. Evident fluctuations in the COF curve of the B01 sample were ascribed to the partially detached region formed after wearing for 50 m, revealing a high COF of 0.72. The transfer layer of the B02 sample formed on a narrow portion of the wear track after accumulating the wear debris. The average COFs in the sliding interval of 30–100 m are listed in Table 3. Both the batch B and C samples exhibited a decreased COF value when prepared at a higher *f*_N2_. For example, the COF values are 0.72, 0.63, and 0.59 for the B01, B02, and B04 samples, respectively. Moreover, the batch C films exhibited much lower COF values than the batch B films. Except for the B01 film, all of the (NbTaMoW)N*_x_* films had wear depths less than their thicknesses, indicating that they were not worn through. The COF curves’ low and smooth variation could be attributed to the MoO_3_ and WO_3_ solid lubricants, reducing debris generation during the wear tests and leading to smooth wear surfaces [40,41]. The wear rate of the C04 film is 3.16 × 10^−7^ mm^3^/Nm, lower than the 8.27 × 10^−7^ and 1.02 × 10^−6^ mm^3^/Nm for the B04 and A04 films. In the films mentioned above, fabricated at an *f*_N2_ of 0.4, the A04 film had the highest hardness, *H*/*E*, and *H*^3^/*E*^2^ ratio, but had the lowest wear resistance. *H*/*E* and *H*^3^/*E*^2^ are the indicators that predict thin films’ toughness and wear resistance [42,43,44], but they do not seem suitable for estimating the performance of laminated films. As reported in previous studies [45,46], the sublayer interfaces in coatings can reduce the COF and improve wear resistance. Multilayer coatings have the characteristics of altering the crack propagation path via the transition zones between adjacent layers [45]. Ref. [47] reported that multilayer coatings exhibited lower hardness and elastic modulus, and higher toughness and adhesion, than monolithic coatings. Moreover, the high H/E ratio and low wear resistance of multilayer films could be attributed to the high intrinsic stress [48]. Figure 10 displays 3D images of the wear tracks examined using white light interferometry, showing smooth tracks. Figure 11 shows the 2D profiles of the cross-section of wear tracks. All (NbTaMoW)N*_x_* films have wear depths less than the thickness, confirming that these films have not been worn through.

## 4. Conclusions

In this study, laminated (NbTaMoW)N*_x_* films were prepared via co-sputtering with different *R_H_* and *f*_N2_ values. The main conclusions are as follows:

(1) With an increasing nitrogen content caused by raising the *f*_N2_, the laminated (NbTaMoW)N*_x_* film structure transitions from multiple BCC phases to a nanocrystalline and FCC phase;

(2) The hardness of laminated (NbTaMoW)N*_x_* films increases with an increase in the nitrogen flow rate. The B04 film had *H* and *E* values of 26.1 GPa and 377 GPa, respectively. Hardness is primarily influenced by the formation of nitride phases and solid solution strengthening, as well as the transition of the bonding structure from metallic to covalent. The formation of a laminated structure influenced the mechanical properties; higher hardness was associated with a decrease in the equilibrated laminated period, which resulted from increased *R_H_* during deposition;

(3) Except for the B01 film, all laminated (NbTaMoW)N*_x_* films had wear depths less than their thicknesses, indicating that they were not worn through. The C04 film showed the lowest wear rate (3.16 × 10^−7^ mm^3^/Nm), which was significantly superior to the B04 and A04 films. In contrast to monolithic coatings, multilayer coatings exhibit lower hardness and increased toughness and wear resistance.

## Figures and Tables

**Table 1:** Sputtering variables for preparing (NbTaMoW)N*_x_* films.

Sample	*R_H_* ^1^ (rpm)	*f*_N2_ ^2^
A01	30	0.1
A02	30	0.2
A04	30	0.4
B01	10	0.1
B02	10	0.2
B04	10	0.4
C01	2	0.1
C02	2	0.2
C03	2	0.4

^1^ *R_H_*: Substrate holder rotation speed. ^2^ *f*_N2_: Nitrogen flow ratio.

**Table 2:** Mechanical properties of (NbTaMoW)N*_x_* films.

Sample	*H* ^a^ (GPa)	*E* ^b^ (GPa)	*H*/*E*	*H*^3^/*E*^2^ (GPa)	*W_e_* ^c^ (%)	Residual Stress (GPa)
A01	24.7 ± 1.3	351 ± 14	0.070	0.122	–	–0.17 ± 0.00
A02	29.3 ± 1.4	333 ± 12	0.088	0.227	–	–0.04 ± 0.01
A04	29.9 ± 0.9	343 ± 8	0.087	0.227	–	–1.14 ± 0.19
B01	21.6 ± 0.5	325 ± 6	0.066	0.095	55	–0.04 ± 0.10
B02	23.0 ± 0.5	337 ± 9	0.068	0.107	57	–0.18 ± 0.04
B04	26.1 ± 0.7	377 ± 16	0.069	0.125	60	–0.91 ± 0.06
C01	19.3 ± 0.9	317 ± 13	0.061	0.071	53	–0.25 ± 0.09
C02	23.4 ± 0.4	337 ± 6	0.069	0.113	58	–0.61 ± 0.21
C04	25.2 ± 1.6	386 ± 23	0.065	0.107	57	–0.80 ± 0.28

^a^ *H*: Hardness. ^b^ *E*: Elastic modulus. ^c^ *W_e_*: Elastic recovery.

**Table 3:** Tribological properties of (NbTaMoW)N*_x_* films.

Sample	Thickness (nm)	Wear Depth (nm)	COF ^a^	Wear Rate (mm^3^/Nm)
A04	1947	86	0.66	1.02 × 10^−6^
B01	3400	110 ^b^	0.72	–
B02	2990	55	0.63	7.67 × 10^−7^
B04	2440	73	0.59	8.27 × 10^−7^
C01	3520	50	0.62	6.18 × 10^−7^
C02	2950	46	0.39	5.70 × 10^−7^
C04	2300	83	0.37	3.16 × 10^−7^

^a^ COF: Coefficient of friction at a sliding distance of 30–100 m. ^b^ Undetached region.

## Data Availability

Data are contained within the article.
